# Outcomes of acute pulmonary embolism in hospitalized patients with cancer

**DOI:** 10.1186/s12890-021-01808-9

**Published:** 2022-01-06

**Authors:** Khalid Shalaby, Adriana Kahn, Elizabeth S. Silver, Min Jung Kim, Kathir Balakumaran, Agnes S. Kim

**Affiliations:** 1 Department of Internal Medicine, University of Connecticut Health Center, 263 Farmington Avenue, Farmington, CT 06030-1235 USA; 2 Advanced Heart Failure and Transplant Cardiology, University Hospitals Cleveland Medical Center, 11100 Euclid Ave, Cleveland, OH 44106 USA; 3 Department of Medicine, Calhoun Cardiology Center, University of Connecticut School of Medicine, 263 Farmington Avenue, Farmington, CT 06030 USA

**Keywords:** Pulmonary embolism, Cancer, Metastasis, Hospitalizations, Mortality

## Abstract

**Background:**

Cancer-associated pulmonary embolism (PE) places a significant burden on patients and health care systems.

**Methods:**

A retrospective cross-sectional analysis of the National Inpatient Sample (NIS) database was performed in patients with acute PE from 2002 to 2014. Among patients hospitalized with PE, we investigated the differences in clinical outcomes and healthcare utilization in patients with and without cancer. A multivariate logistic regression model was applied to calculate adjusted odds ratios (OR) to estimate the impact of cancer on clinical outcomes. Wilcoxon rank sum tests were used to determine the differences in healthcare utilization between the two cohorts.

**Results:**

Among 3,313,044 patients who were discharged with a diagnosis of acute PE, 84.2% did not have cancer, while 15.8% had cancer as a comorbidity (56% metastatic cancer, 35% solid tumor without metastasis, and 9% lymphoma). Patients with cancer had a higher mean age but lower rates of common comorbidities except for coagulation deficiency than patients without a cancer diagnosis. In patients with cancer, the rate of IVC filter placement was higher (21.7% vs. 13.11%, OR 1.76 (95% CI 1.73–1.79); p < 0.0001) and thrombolytic use lower (1.34% vs. 2.15%, OR 0.68 (95% CI 0.64–0.72); p < 0.0001). Patients with cancer hospitalized for PE had a higher all-cause in-hospital mortality (11.8% vs. 6.6%, OR 1.79 (95% CI 1.75–1.83); p < 0.0001), longer length of stay (6 vs. 5 days; p < 0.0001), higher total charge per hospitalization ($30,885 vs. $27,273; p < 0.0001), and higher rates of home health services upon discharge (35.8% vs. 23.2%; p < 0.0001) compared with those without cancer.

**Conclusion:**

Concurrent cancer diagnosis in patients hospitalized for acute PE was associated with a 90% increase in all-cause mortality, longer length of stay, higher total charge per hospitalization, and higher rates of home health services upon discharge. The majority (56%) of patients with cancer had metastatic disease. Furthermore, there were identifiable differences in the intervention for acute PE between the two groups.

## Introduction

The annual incidence of acute pulmonary embolism (PE) in the USA has been increasing over the past two decades because of a longer life expectancy and improvement in diagnostic imaging tests [1, 2]. Concomitantly, inpatient admissions and hospital charges for PE have been rising [3, 4].

Cancer is a well-known risk factor for the development of venous thromboembolism (VTE) [5, 6]. Venous stasis, endothelial injury, and hypercoagulable state (Virchow’s triad) play a role. The incidence of VTE in patients with cancer varies among studies depending on the type and stage of cancer, treatment exposure, duration of follow-up, and method of detecting and reporting thrombotic events [5, 6]. There is an association between cancer aggressiveness and thrombogenesis, with metastatic disease being described as one of the strongest predictors of VTE [5, 7]. There has been a recent increase in the incidence of VTE among patients with cancer [5, 6].

Although multiple studies report the burden of PE in the general population and the increased risk of VTE in patients with cancer separately, there is a lack of recent data comparing the outcomes of PE in the presence or absence of cancer in the inpatient setting on a national scale in the USA. The National Inpatient Sample Healthcare Cost and Utilization Project (NIS HCUP) database is one of the largest available all-payer databases that includes data on more than 7 million hospital admissions each year, which when weighted reflects on a population of 35 million hospital admissions across different geographical areas in the USA and therefore may represent a significant sample of clinically relevant PE hospitalizations in patients with cancer.

The objective of this study was to investigate the clinical and healthcare utilization outcomes of patients hospitalized with acute PE in the presence or absence of cancer as a comorbidity.

## Methods

We queried the National Inpatient Sample (NIS) database from the Healthcare Cost and Utilization (HCUP) Project in Agency for Healthcare Research and Quality (AHRQ) for hospitalizations between 2002 and 2014 where the primary or secondary diagnosis was pulmonary embolism using International Classification of Diseases, 9th revision (ICD-9) codes. We utilized ICD-9 codes for saddle pulmonary embolism (415.13) and other pulmonary embolism (415.19) as our primary studied population. Since the database does not contain any patients’ identifiers, it was exempt from Institutional Review Board.

All statistics incorporated discharge-level weights provided by the NIS database in order to account for the variation of sampling. NIS is a 20% stratified sample of all discharges from the USA and sampled by hospitals rather than individuals. Clustering of records within hospitals was accounted. Given the variability in the contribution by hospital to the sample, we applied the sample weights to calculate the national estimates for the trend analysis appropriately.

We divided our primary studied population into a group with a diagnosis of cancer as a comorbidity and a group without a cancer diagnosis. We defined cancer as a comorbidity if either of the following three Elixhauser comorbidity measures (lymphoma, metastatic cancer, and solid tumor without metastasis) were present in the same hospitalization. Each Elixhauser comorbidity measure comprises a comprehensive list of ICD-9 codes of the disease category. A list of ICD-9 codes under each variable is publicly available on HCUP website. https://www.hcupus.ahrq.gov/toolssoftware/comorbidity/Table2-FY12-V3_7.pdf.

We excluded the following ICD-9 codes from our analysis; iatrogenic PE (415.11), septic PE (415.12), primary pulmonary hypertension (416.0), kyphoscoliotic heart disease (416.1), chronic PE (416.2), chronic pulmonary heart disease (416.8, 416.9), AV fistula pulmonary vessel (417.0), pulmonary artery aneurysm (417.1), pulmonary circulatory disease (417.8), pulmonary circulatory disease NOS (417.9) and history of PE (V12.55). We also excluded other obstetrical pulmonary embolism, unspecified as to episode of care or not applicable (673.80), other obstetrical pulmonary embolism, delivered, with or without mention of antepartum condition (673.81), other obstetrical pulmonary embolism, delivered, with mention of postpartum complication (673.82), other obstetrical pulmonary embolism, antepartum condition or complication (673.83), and other obstetrical pulmonary embolism, postpartum condition or complication (673.84).

We compared differences in baseline characteristics of our two studied groups including differences in age, race, and gender breakdown as well as insurance coverage. We identified differences in baseline comorbidities between the two groups using other categories of the 29 Elixhauser comorbidity measures including hypertension, diabetes mellitus, diabetes mellitus with complications, heart failure, renal failure, obesity, paralysis, and coagulation deficiency.

All-cause in-hospital mortality was the primary outcome studied in both groups. We compared the rates of clinical outcomes during the same hospitalization, which included the use of invasive mechanical ventilation (procedure codes 96.70, 96.71, 96.72), non-septic shock (ICD-9 codes 785.50, 785.51, 785.59), vasopressor use (procedure code 00.17), thrombolytics injections (procedure code 99.10), and IVC filter placements (procedure code 38.7). Total length of stay (LOS), hospitalization charges in US dollars, AHRQ Elixhauser risk of readmission score, and disposition on discharge were also compared across the two groups. Elixhauser readmission score was calculated as the weighted sum of 29 individual comorbidities as per Moore et al. [8].

All statistical analyses were performed using the weighted survey methods in SAS (version 9.4, SAS Institute Inc., Cary, NC). The data were visually inspected and tested for normality using Kolmogorov–Smirnov test to determine appropriate statistical approaches. Descriptive summary statistics for baseline characteristics were presented as frequencies with percentages for categorical variables and were compared using a Pearson’s χ^2^ test and Fisher’s exact test to compare the admission cases between groups with and without cancer comorbidity. All summary statistics for continuous variables were reported as means with standard deviation (SD) for normally distributed continuous variables and two-sample t-test was used to compare the two PE groups with and without cancer comorbidity. If variables were non-normally distributed continuous data, variables were presented as medians and interquartile range (IQR), and two-sample Wilcoxon rank sum tests were applied for comparisons. All potential confounders (patient characteristics and comorbidities) with p value < 0.05 in univariate logistic regressions were included in multivariate logistic models, which were used to calculate the adjusted odds ratios (OR) with 95% confidence intervals (95% CIs) to estimate the impact of cancer on clinical outcomes. We set statistical significance at an alpha level of 0.05.

## Results

**Fig. 1:**
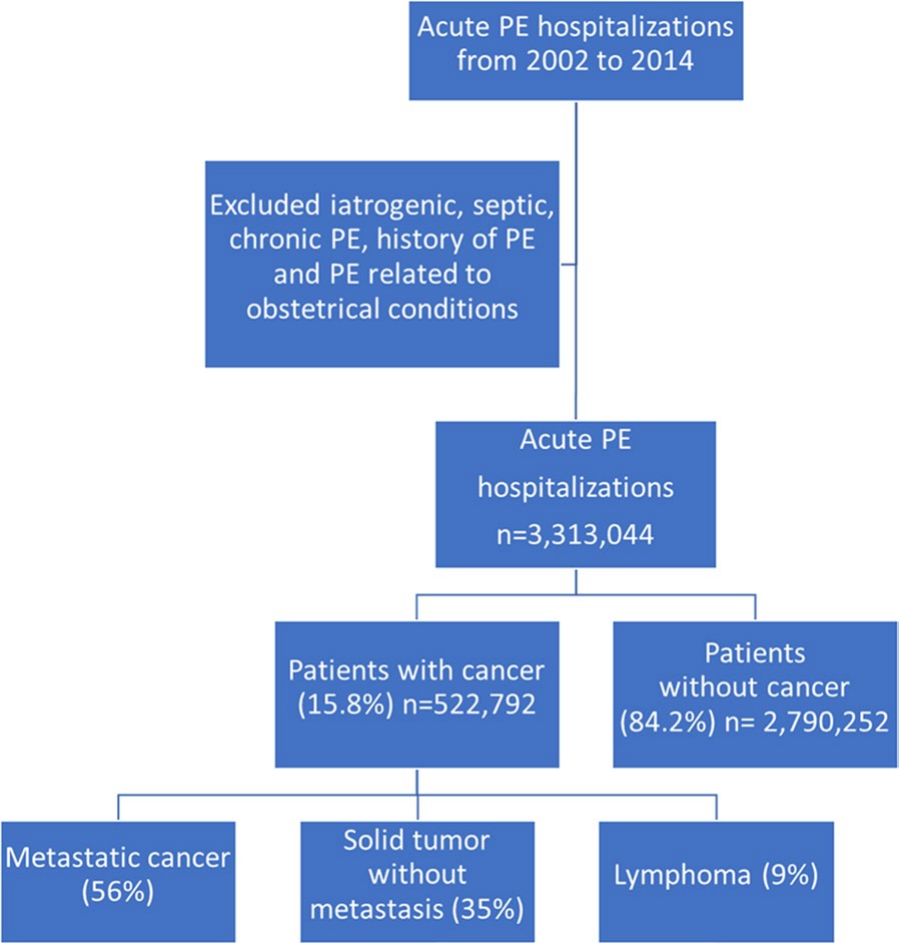
Acute PE hospitalizations from 2002 to 2014 with and without cancer diagnosis

There were approximately 671,852 patients (national weighted estimates: 3,313,044) discharged with a diagnosis of acute PE between the years 2002–2014. Among these patients, 566,917 (national weighted estimates: 2,790,252; 84.2%) did not have a concurrent diagnosis of cancer, and 105,935 (national weighted estimates: 522,792;15.8%) had cancer as a comorbidity (Fig. 1). Among the patients with cancer, 56% had metastatic cancer, 35% had solid tumor without metastasis, and 9% had lymphoma (Fig. 1).

The mean age of patients with cancer was 66.2 years, whereas the mean age of patients without cancer was 63.1 years, p < 0.0001 (Table 1). Female gender was predominant in both patient groups with and without cancer (Table 1). White race constituted the majority in both groups (Table 1). Medicare was the most common payer in both groups, followed by private insurance, then Medicaid (Table 1).

**Table 1:** Demographics and baseline characteristics

Patient characteristics	Patients with cancer	Patients without cancer	Overall
Age*			
Years: Mean ± Std	66.20 ± 12.97	63.06 ± 17.73	63.55 ± 17.10
**Gender**			
Male	48.5%	46.1%	46.5%
Female	51.5%	53.9%	53.5%
**Race**			
White	75.8%	74.0%	74.3%
Black	14.5%	16.7%	16.3%
Hispanic	5.6%	5.7%	5.7%
Asian or Pacific Islander	1.5%	1.0%	1.0%
Native American and Others	2.6%	2.7%	2.6%
**Primary payer**			
Medicare	56.1%	53.2%	53.7%
Medicaid	8.2%	9.0%	8.9%
Private insurance	31.2%	29.8%	30.0%
Self-pay	2.1%	4.5%	4.2%
No-charge/other	2.4%	3.5%	3.3%

Demographics and characteristics of hospitalized PE patients with and without cancer diagnosis

*P < 0.0001

**Fig. 2:**
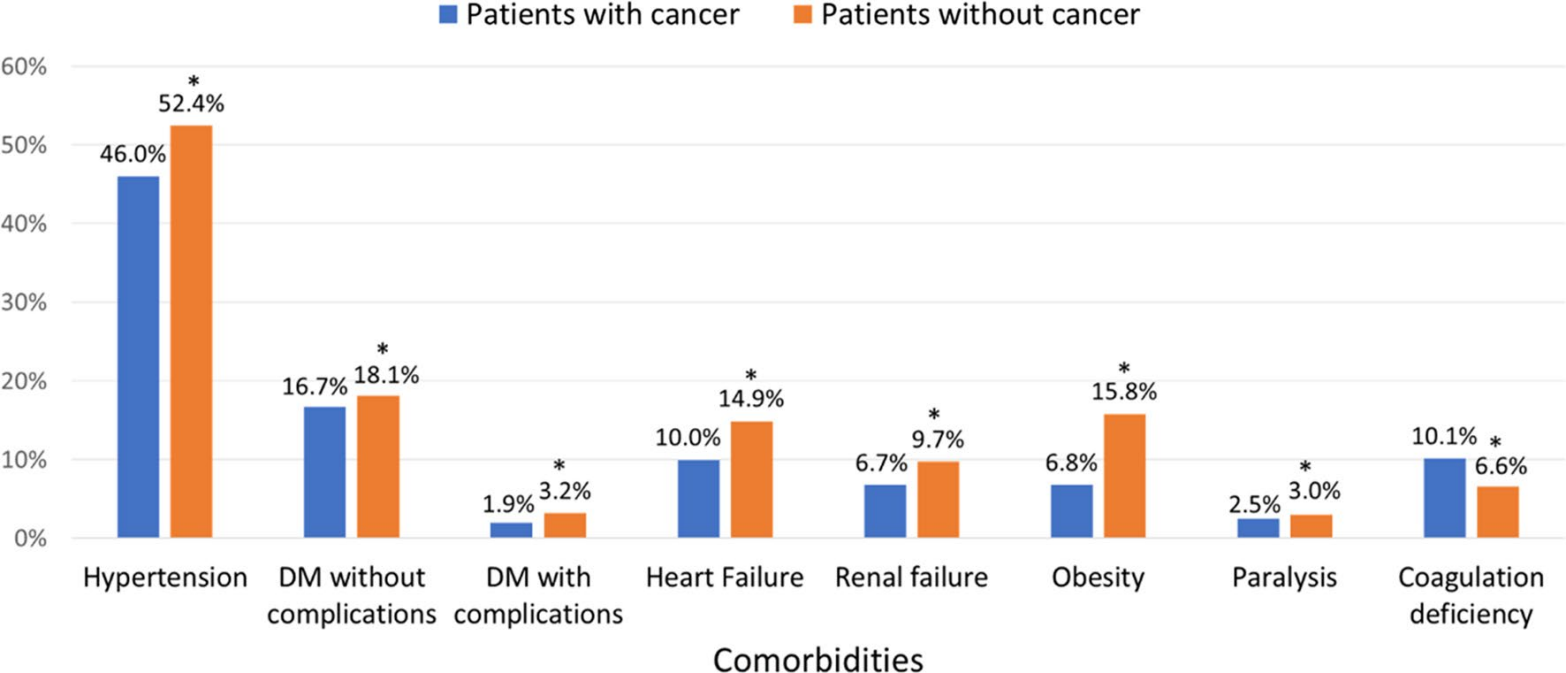
Rates of comorbidities in hospitalized PE patients with and without cancer diagnosis, *P < 0.0001. *DM* diabetes mellitus

The rates of common chronic non-communicable comorbidities using Elixhauser comorbidity variables were predominantly lower in patients with cancer than in those without, including hypertension (46% vs. 52.4%; p < 0.0001), diabetes mellitus without complications (16.7% vs. 18.1%; p < 0.0001), diabetes mellitus with complications (1.9% vs. 3.2%; p < 0.0001), heart failure (10% vs. 14.9%; P < 0.0001), renal failure (6.7% vs. 9.7%; p < 0.0001), obesity (6.8% vs. 15.8%; p < 0.0001) and paralysis (2.5% vs. 3%; p < 0.0001). The exception to the above pattern was the rate of co-existent coagulation deficiency, which was higher in patients with cancer (10.1% vs. 6.6%; p < 0.0001) (Fig. 2).

The in-hospital all-cause mortality was significantly higher in patients with cancer than in those without (11.8% vs. 6.6%, OR 1.79, 95% CI 1.75–1.83; p < 0.0001) (Table 2). The rate of thrombolytic administration was lower in patients with cancer (1.34% vs. 2.15%, OR 0.68, 95% CI 0.64–0.72; p < 0.0001), while the rate of IVC filter placement was significantly higher in the same group (21.71% vs. 13.11%, OR 1.76, 95% CI 1.73–1.79; p < 0.0001). The rate of invasive mechanical ventilation was marginally but significantly lower in patients with cancer (7.06% vs. 7.23%, OR 0.95, 95% CI 0.92–0.97; p < 0.0001). Despite lower rates of non-septic shock in patients with cancer (1.72% vs. 1.84%, OR 0.90, 95% CI 0.86–0.95; p < 0.0001), vasopressor use was greater in the same group (0.66% vs. 0.51%, OR 1.25, 95% CI 1.15–1.36; p < 0.0001) (Table 2).

**Table 2:** Clinical outcomes and interventions

	Patients with cancer (%)	Patients without cancer (%)	OR (95% CI)
Inpatient mortality*	11.80	6.60	1.79 (1.75–1.83)
Thrombolytic injection*	1.34	2.15	0.68 (0.64–0.72)
IVC filter placement*	21.71	13.11	1.76 (1.73–1.79)
Invasive mechanical ventilation*	7.06	7.23	0.95 (0.92–0.97)
Vasopressor use*	0.66	0.51	1.25 (1.15–1.36)
Shock (non-septic) *	1.72	1.84	0.90 (0.86–0.95)

Clinical outcomes and interventions in hospitalized PE patients with and without cancer

IVC: Inferior Vena Cava, OR: Odds Ratio, CI: Confidence Interval

^*^ P < 0.0001

Among hospitalizations for acute PE, patients with cancer had a significantly longer length of stay with a median of 6 days (IQR 3–10) vs. 5 days (IQR 3–9; p < 0.0001) and higher median total charges per hospitalization 30,885 USD (IQR 16,308–61,113; p < 0.0001) compared with 27,273 USD (IQR 15,271–53,340) in patients without cancer (Table 3). The assessed risk of readmission was also higher in patients with cancer, with a higher median AHRQ Elixhauser risk of readmission score of 31 (IQR 23–41) vs. 12 (IQR 4–22; p < 0.0001) (Table 3).

**Table 3:** Healthcare cost and utilization metrics

Healthcare cost and utilization: median (IQR)	Patients with cancer	Patients without cancer
Length of stay* (days)	6 (3–10)	5 (3–9)
Total charges per hospitalization* (USD)	$ 30,885 (16,308–61,113)	$ 27,273 (15,271–53,340)
AHRQ Elixhauser risk of readmission score*	31 (23–41)	12 (4–22)

Healthcare cost and utilization metrics in hospitalized PE patients with and without cancer

*IQR* interquartile range, *USD* US dollar, *AHRQ* Agency for Healthcare Research and Quality

*P < 0.0001

A higher proportion of patients with cancer was discharged with home health services including home hospice (Table 4).

**Table 4:** Disposition

Disposition	Patients with cancer (%)	Patients without cancer (%)	Overall (%)
Routine: home or self-care*	43.8	53.9	52.3
Transfer to short-term hospital*	2.8	3.3	3.2
Transfer other*^†^	17.6	19.7	19.3
Home health care*^‡^	35.8	23.2	25.2

Disposition at the time of discharge of hospitalized PE patients with and without cancer

*P < 0.0001

^†^Includes skilled nursing facility, intermediate care facility, inpatient rehab facility, hospice facility

^‡^Includes home health services, home hospice

## Discussion

PE constitutes significant morbidity and mortality in patients with cancer requiring hospitalization. VTE has been reported as the second most common cause of death in patients with cancer [5, 6]. To the best of our knowledge, this is the largest cross-sectional analysis of patients with and without cancer admitted for acute PE utilizing the NIS database, which allowed the comparison of differences in outcomes between the two groups.

Our demographic results show an older population in patients with cancer. Advanced age has been shown to be independently associated with poorer outcomes, including greater inpatient mortality and length of stay [3]. Cancer is largely a disease of older age, and the geriatric population has expanded in the recent decades [9]. When a multivariate logistic regression model was performed to adjust for the confounders of age and comorbidities, the adjusted odds ratios remained highly significant, indicating that cancer is an independent predictor of worse clinical outcomes. We also observed a higher proportion of female patients among both groups. While some studies have demonstrated a higher incidence of VTE in men, others revealed no difference or higher incidence in women [10–12].

We did not observe major differences between the groups in terms of race distribution. A higher incidence of VTE has been reported among Caucasians and African Americans than among Hispanic persons and Asian-Pacific Islanders [12], and our results are consistent with this finding. The percentage of Medicare coverage in patients with cancer is almost 3% higher, which is consistent with a higher mean age.

Interestingly, the rates of comorbidities differed between the two groups. Obesity was less frequent in patients with cancer presenting with PE. This was surprising since multiple cancers have been identified as obesity-associated tumors, including mammary, renal, esophageal, gastrointestinal, and reproductive cancers in both men and women [13]. A possible explanation is that cancers that are highly associated with PE (such as pancreas, brain, lung, and ovarian cancer) are as a group not directly associated with obesity. Additionally, most of the patients in the cancer group had metastatic disease reflecting a later stage of cancer with cancer-related cachexia possibly contributing to lower body mass index [5]. Similarly, the rates of hypertension, diabetes mellitus, renal failure, and congestive heart failure were lower in patients with cancer than those without. The rates of traditional PE risk factors (e.g. obesity and paralysis) were lower in patients with cancer, suggesting that cancer and/or its treatment is the main risk factor for acute PE in our studied group.

The overall rate of IVC filter placement in acute PE hospitalizations is consistent with previous studies at 14.5% in our study [14]. Patients with cancer received more IVC filter procedures than those without, which may be related to the higher rates of coagulation deficiency in the cancer group along with the overall higher rates of PE recurrence in patients with cancer [15]. The rate of thrombolytic administration was lower in patients with cancer than those without. Possible explanations include less frequent occurrence of massive PE necessitating thrombolytics, which may be reflected by lower rates of non-septic shock in patients with cancer, as well as higher rates of coagulation deficiency (which includes thrombocytopenia and bleeding due to anticoagulation) that could restrict the use of thrombolytics. While the rate of mechanical ventilation was marginally lower in patients with cancer, it does not seem to be clinically significant in the larger context of our interpretation of the data. It may also be related to more conservative approach (Do Not Intubate status) that aligns with the goals of care in patients with advanced cancer. Higher rates of vasopressor use in the setting of lower rates of non-septic shock suggest higher rates of infection and septic shock in patients with cancer. More prospective studies are needed to further elucidate these differences.

Cancer diagnosis was associated with a higher inpatient all-cause mortality in PE hospitalizations. Given the limitations of the database, we are unable to identify if the higher mortality is directly related to the cardiopulmonary complications of PE, cancer progression, or other causes. The estimated risk of readmission in patients with cancer was higher despite a higher proportion of this group receiving home health services, including home hospice, at the time of discharge, indicating the clinical vulnerability of patients with cancer and PE. In an older study, higher readmission rates were observed in patients with concomitant VTE and cancer diagnosis compared with either diagnosis solely [16].

Patients with cancer hospitalized with acute PE had a longer length of stay. Studies show that direct-acting oral anticoagulants are associated with a shorter length of stay and lower hospitalization costs when compared with warfarin [17]. Between 2002 and 2014, our studied period, newer oral anticoagulants were not considered standard of care for patients with cancer, who were historically anticoagulated with low molecular weight heparin or warfarin, which requires waiting 3–5 days to achieve a therapeutic level. Longer length of stay contributes to the higher total charge per hospitalization. Due to the limitation of this database, we are unable to discern whether other diagnostic or therapeutic interventions during the hospital stay may have increased the total cost.

Limitations of the study include the weaknesses of the database itself in that data points are extracted from deidentified hospitalizations rather than individual patient charts. We realize there is a large degree of heterogeneity among patients with cancer as not all malignancies carry equal risks of thromboembolism. The group of patients with cancer included patients with the 3 Elixhauser comorbidity measures (lymphoma, metastatic cancer, and solid tumor without metastasis) used as a filter. It did not include patients with leukemia, who remain at risk of PE. Additionally, there is no post-discharge data on morbidity and mortality for these hospitalizations. Our studied population included patients with a PE diagnosis on admission and hospital-acquired PE. We could not differentiate between these two groups.

## Conclusions

Concurrent cancer diagnosis in patients hospitalized for acute PE between 2002 and 2014 was associated with a 90% increase in all-cause inpatient mortality, longer length of stay, higher total charge per hospitalization, higher risk of readmission, and higher rates of home health services upon discharge. The risk factors that drive poor outcomes among patients with cancer and PE remain to be determined. Early identification of oncology patients at the highest risk for VTE and worst outcomes has the potential to decrease morbidity and mortality. The utility and safety of prophylactic anticoagulation in patients at the highest risk for poor PE outcomes remain unknown. It is imperative for clinicians and healthcare systems to implement changes to reduce the length of stay and mitigate the risk of readmission in patients with cancer. This special cohort may require a higher level of clinical care compared with other individuals with PE. The majority (56%) of patients with cancer who were hospitalized with acute PE had metastatic disease. There were identifiable differences in the intervention for acute PE between the two groups, such as rates of IVC filter placement and thrombolytic therapy. Over 1 in 5 patients with cancer received an IVC filter. The safety and efficacy of thrombolytic therapy in patients with cancer, stratified by the type of cancer, should be further investigated. In addition, future studies are needed to assess the impact of direct oral anticoagulants, which are now widely used for the treatment of VTE, on the outcomes of PE hospitalizations in patients with and without cancer. This study inspires more research to determine the optimal strategies for prevention and management of VTE in patients with cancer.

## Data Availability

The datasets generated and/or analyzed during the current study are not publicly available but are available for purchase through the HCUP Central distributor. https://www.hcup-us.ahrq.gov/tech_assist/centdist.jsp.

## Abbreviations

PE: Pulmonary embolism
VTE: Venous Thromboembolism
NIS: National Inpatient Sample
HCUP: Healthcare Cost and Utilization Project
AHRQ: Agency for Healthcare Research and Quality
ICD: International Classification of Diseases
IQR: Interquartile Range
NOS: Not Otherwise Specified
IVC: Inferior Vena Cava
LOS: Length of Stay
